# Hypoxia regulates human lung fibroblast proliferation via p53-dependent and -independent pathways

**DOI:** 10.1186/1465-9921-10-17

**Published:** 2009-03-06

**Authors:** Shiro Mizuno, Herman J Bogaard, Norbert F Voelkel, Yukihiro Umeda, Maiko Kadowaki, Shingo Ameshima, Isamu Miyamori, Takeshi Ishizaki

**Affiliations:** 1 Third Department of Internal Medicine, University of Fukui, 23-3 Eiheiji-cho, Matsuoka, Yoshida-gun, Fukui, Japan; 2 Pulmonary and Critical Care Medicine Division, Virginia Commonwealth University, 1101 E Marshall St, Sanger Hall 7th Floor, Room 7-020, Richmond, Virginia, USA; 3 Department of Fundamental Nursing, University of Fukui, 23-3 Eiheiji-cho, Matsuoka, Yoshida-gun, Fukui, Japan

## Abstract

**Background:**

Hypoxia induces the proliferation of lung fibroblasts in vivo and in vitro. However, the subcellular interactions between hypoxia and expression of tumor suppressor p53 and cyclin-dependent kinase inhibitors p21 and p27 remain unclear.

**Methods:**

Normal human lung fibroblasts (NHLF) were cultured in a hypoxic chamber or exposed to desferroxamine (DFX). DNA synthesis was measured using bromodeoxyuridine incorporation, and expression of p53, p21 and p27 was measured using real-time RT-PCR and Western blot analysis.

**Results:**

DNA synthesis was increased by moderate hypoxia (2% oxygen) but was decreased by severe hypoxia (0.1% oxygen) and DFX. Moderate hypoxia decreased p21 synthesis without affecting p53 synthesis, whereas severe hypoxia and DFX increased synthesis of both p21 and p53. p27 protein expression was decreased by severe hypoxia and DFX. Gene silencing of p21 and p27 promoted DNA synthesis at ambient oxygen concentrations. p21 and p53 gene silencing lessened the decrease in DNA synthesis due to severe hypoxia or DFX exposure. p21 gene silencing prevented increased DNA synthesis in moderate hypoxia. p27 protein expression was significantly increased by p53 gene silencing, and was decreased by wild-type p53 gene transfection.

**Conclusion:**

These results indicate that in NHLF, severe hypoxia leads to cell cycle arrest via the p53-p21 pathway, but that moderate hypoxia enhances cell proliferation via the p21 pathway in a p53-independent manner. In addition, our results suggest that p27 may be involved in compensating for p53 in cultured NHLF proliferation.

## Background

Hypoxia is observed in many physiological and pathological conditions, including interstitial lung diseases, acute respiratory distress syndrome, chronic obstructive pulmonary diseases, asthma, wounded tissues, neoplasmas, and atherosclerosis [1-5]. Under such hypoxic conditions, fibroblast proliferation with enhanced production of extracellular matrix (ECM) and marked fibrosis are key components to understanding tissue remodeling [6,7]. Fibroblast proliferation with enhanced production of ECM is an important feature of hypoxia-associated lung diseases, and several in vitro studies have also shown that exposure to moderate hypoxia stimulates the proliferation of lung fibroblasts, with enhanced production of collagen molecules [2,8,9]. In addition, hypoxia is one of the factors known to cause secondary pulmonary hypertension and pulmonary vascular remodeling [2]. According to a WHO statement in 1996, there were approximately 140 million people living at altitudes above 2500 m and there are several areas of permanent habitation at altitudes in excess of 4000 m. After several weeks of exposure to high altitude, lowlanders develop pulmonary hypertension, which is not completely reversed by supplemental oxygen [10], suggesting development of vascular remodeling of the lung [11]. Secondary pulmonary hypertension is characterized by proliferation of vascular smooth muscle cells and pulmonary arterial fibroblasts with enhanced deposition of ECM in small pulmonary vessels [12-14]. These results suggest that hypoxic enhancement of lung fibroblast proliferation contributes to the deposition of collagen fibrils in the lung and progression of hypoxia-associated lung diseases.

Under normal physiological conditions, the majority of pulmonary cells are in a quiescent state, so for proliferation of pulmonary cells, which underlies pulmonary remodeling, cells must enter the cell cycle. The most important molecular event necessary for progress of the cell cycle is phosphorylation of the retinoblastoma protein by cyclin-dependent kinase (CDK)-cyclin complexes [15]. CDK activity can be inhibited by CDK inhibitors (CKI) such as p21 and p27. Up-regulation of CKI blocks cell cycle progression in the G1 phase, and down-regulation of CKI is required for entry into the S phase [7]. However, the effect of hypoxia on mammalian cell proliferation seems to be dependent on the cell type and on oxygen concentration. In several cell types, severe hypoxia or chemically induced anoxia has been shown to induce G1 cell cycle arrest [16,17], whereas moderate hypoxia has been shown to enhance cell proliferation [3,18,19]. The results of previous studies have suggested that p21 plays an important role in oxygen-dependent cell proliferation [20,21], and that p27 regulates both hypoxic pulmonary remodeling and cell cycle arrest in severe hypoxia [17,22-25].

CKI p21 is a key regulator of the cell cycle when cells are exposed to oxidative stress or NO, and plays an important role in pulmonary arterial smooth muscle cell (PASMC) proliferation via induction of p53 [26,27]. In tumors expressing wild-type p53, the locations of cells undergoing apoptosis strongly correlate with regions of hypoxia, whereas tumors expressing mutant p53 have lower levels of apoptosis in hypoxic regions [28]. p53 knock-out mutant cells are more resistant to hypoxia-induced apoptosis, and have a selective growth advantage compared with wild-type p53 cells [28,29]. These results support the view that p53 has opposite functions toward cell proliferation under hypoxia. In addition, p53 accumulation under hypoxic conditions is linked to hypoxia inducible factor-1α (HIF-1α), which is known to be a central transcriptional factor operating during hypoxia toward angiogenesis [30,31].

Given these previous findings, it seems likely that p21, p27 and p53 are key mediators in the hypoxic proliferation of lung fibroblasts. However, little is known about the interactions between these proteins in this situation. Moreover, it is also uncertain whether hypoxic accumulation of p53 induces functional p21 protein [14,32-34], since severe hypoxia and anoxia have been reported to induce p53 protein by a pathway different from the pathway induced by DNA-damaging agents [35]. The purpose of this study was to investigate the participation of and interaction between p21, p27 and p53 with respect to the effect of hypoxic pulmonary proliferation in cultured normal human lung fibroblasts (NHLF) during hypoxia, and with respect to anoxia chemically induced by desferroxamine (DFX). DFX is known as an iron chelator which depletes iron and inhibits oxygen, Fe^2+-^, and oxoglutarate-dependent dioxygenases [36].

## Methods

### Chemicals

Chemicals and materials were obtained from the following sources: FGM-2 medium, recombinant human fibroblast growth factor (FGF), gentamycin, streptomycin and amphotericin B came from Sanko Junyaku Co., Ltd. (Tokyo, Japan); the bromodeoxyuridine (BrdU) proliferation assay kit came from OncogeneTM (Cambridge, MA, USA); the ECL system came from Amersham (Buckinghamshire, UK); Moloney murine leukemia virus reverse transcriptase came from Toyobo Co. Ltd. (Osaka, Japan); the QuantitechTM SYBR Green PCR kit came from Qiagen (Santa Clarita, CA, USA); Lipofectamine 2000, 4–12% Bis-Tris Nupage gels, and MES-SDS running buffer came from Invitrogen (Carlsbad, CA, USA); the DC protein assay kit and polyvinylidene difluoride (PVDF) membranes came from Bio-Rad Laboratories (Richmond, CA, USA); rabbit anti-p21 polyclonal antibody, rabbit anti-p27 polyclonal antibody, mouse anti-p53 monoclonal antibody, mouse anti-β-actin monoclonal antibody, and horseradish peroxidase-conjugated goat anti-mouse and rabbit came from Santa Cruz Biotechnology Inc. (Santa Cruz, CA, USA). All other chemicals were purchased from Sigma (St. Louis, MO, USA).

### Cell cultures

NHLF were supplied by Sankou Junyaku Co., Ltd. and grown in FGM-2 medium containing 2% fetal bovine serum (FBS) with 50 μg/ml gentamycin, 50 ng/ml amphotericin B and 1 ng/ml recombinant human FGF. Cells were cultured in 75-cm^2 ^tissue culture flasks (Corning, NY, USA) in a cell-culture incubator (37°C, 5% CO_2_, and 95% air), and used after the seventh passage following trypsinization. According to the manufacture's data sheet, NHLF are obtained from human peripheral lung tissue with enzymatic digestion, and the cells are confirmed negative staining for smooth muscle α-actin, cytokeratin 18, cytokeratin 19, and von Willebrand factor. The light microscopic appearance of NHLF was unchanged and we confirmed negative staining of smooth muscle α-actin up to seventh passage, which excluded the transformation of NHLF into a myofibroblast phenotype. Oxygen concentrations (0.1 – 10%) were modified using N_2_-CO_2 _incubators (BNR-110M; Tabai ESPEC Corp., Tokyo, Japan; 10–0233, Ikemoto Rika Kogyo, Co., Ltd., Tokyo, Japan).

### Assay of BrdU incorporation by NHLF

NHLF were seeded in 96-well culture disks at a density of 6,000 cells/cm^2 ^and incubated for 48 h in serum-free Dulbecco's modified Eagle's medium (DMEM), after which the medium was changed to DMEM with 10% FBS and antibiotics. The cells were then incubated for another 24 h in various oxygen concentrations, and various concentrations of DFX were added to some cultures. BrdU incorporation was measured using a BrdU proliferation assay kit in accordance with the manufacturer's protocol. Briefly, the cells were labeled with 10 ng/ml BrdU during incubation. After labeling, the cells were washed three times with cold phosphate-buffered saline (PBS), fixed, air dried and treated with mouse anti-BrdU monoclonal antibody (1:1000). After aspiration of the antibody solution, the cells were washed three times and incubated with peroxidase goat anti-mouse IgG (1:2000) at room temperature for 30 min, then the cells were washed three times and 100 μM substrate solution was added to each well. Plates were incubated for 10 min in the dark, after which dual-wavelength absorbance at 450 – 540 nm was measured.

### Propidium iodide staining

To determine whether the cell cycle is influenced by oxygen concentration, flow cytometric analysis with propidium iodide staining was performed. NHLF were seeded into 6-well culture disks at a density of 6,000 cells/cm^2 ^and incubated for 48 h in serum-free DMEM, after which the medium was changed to DMEM with 10% FBS and antibiotics. Next, the cells were incubated for 24 h in various concentrations of oxygen. To measure the DNA content, the cells were harvested using trypsin and ethylenediamine tetraacetic acid (EDTA) and fixed with 70% ethanol. The ethanol was removed and cells were incubated in PBS containing RNase (172 kunits/ml) at 37°C for 30 min, stained with propidium iodide (50 μg/ml), and suspended in PBS for 30 min on ice. DNA fluorescence was measured and flow cytometric analyses were performed using an EPICS XL flow cytometer (Beckman Coulter, CA, USA).

### Real-time RT-PCR analysis of p21, p27, and p53 mRNA

NHLF were cultured in 6-well flat-bottomed culture plates at a density of 6,000 cells/cm^2 ^and cultured for 48 h in serum free DMEM. The cells were washed twice with PBS, then placed in DMEM supplemented with 10% FBS and antibiotics in various concentrations of oxygen for the indicated times with or without 100 μM DFX. The cells were then harvested by trypsinization, washed three times, and pelleted by centrifugation. Total cellular RNA was obtained from the cells by a single extraction with an acid guanidinium thiocyanate-phenol-chloroform mixture [26]. RT was performed using 0.5 μg of total RNA. cDNA synthesis was performed using 200 U of Moloney murine leukemia virus reverse transcriptase, 5 μM oligoDT, 1 mM dNTP solution, and 3 mM Mg^2+ ^in a volume of 20 μl. The temperature profile was comprised of annealing at room temperature for 5 min, extension at 44°C for 40 min, and termination at 99°C for 5 min. PCR was performed for the resulting RT products using oligonucleotide primers specific for p21, p27, p53, and β-actin. The primers used were as follows: p21 forward primer 5'-GGAAGACCATGTGGACCTGT-3', reverse primer 5'-GGCGTTTGGAGTGGTAGAAA-3'; p27 forward primer 5'-GCCCTCCCCAGTCTCTCTTA-3', reverse primer 5'-TCAAAACTCCCAAGCACCTC-3'; p53 forward primer 5'-GTTCCGAGAGCTGAATGAGG-3', reverse primer 5'-TTATGGCGGGAGGTAGACTG-3'; β-actin forward primer 5'-GCAAGCAGGAGTATGACGAG-3', reverse primer 5'-CAAATAAAGCCATGCCAATC-3'. All PCR reactions were performed with a LightCycler™ PCR system (Roche Diagnostics, Meylan, France) using DNA-binding SYBR Green dye for detection of the PCR products. The cycling conditions were as follows: initial denaturation at 95°C for 15 min, followed by 50 cycles of denaturation at 94°C for 15 s, annealing at 55°C for 15 s, and extension at 72°C for 15 s. The β-actin gene was used as a reference. The PCR products were isolated from the LightCycler™ glass capillaries and visualized by electrophoresis on 1.5% agarose gels followed by ethidium bromide staining to confirm the products. Each assay was replicated in six independent experiments.

### Analysis of p21 mRNA stability

NHLF (6,000 cells/cm^2^) were seeded into 6-cm dishes for 48 h in serum-free DMEM. The cells were washed twice with PBS, then placed in DMEM containing 10% FBS and cultured under various oxygen concentrations for the indicated periods with or without 100 μM DFX in the presence of the transcription inhibitor actinomycin D (Act D) (400 nM). The cells were then counted and p21 mRNA stability was examined per 50,000 cells incubated with Act D using real-time RT-PCR. Each assay was replicated in six independent experiments.

### Western blot analysis

NHLF were seeded into 10-cm dishes at a density of 6,000 cells/cm^2 ^and cultured for 48 h in serum-free DMEM. The cells were washed twice with PBS, then placed in DMEM supplemented with 10% FBS and antibiotics. Cells were then cultured in various oxygen concentrations for the indicated times with or without 100 μM DFX. After incubation, the cells were harvested and resuspended in protein lysis buffer (150 mM NaCl, 20 mM Tris-HCl, 1% NP40, 10 mM EDTA, 10% glycerol, 1 mM PMSF, 10 μg/ml aprotinin, 1 μg/ml leupeptin, 1 μg/ml pepstatin), then incubated for 30 min on ice. After incubation, the cell lysis buffers were centrifuged at 10,000 g for 15 min at 4°C to remove the cell fragments, and the supernatants were analyzed for protein content using a DC protein assay kit. Each sample was quantified, and then 25 μg of protein was loaded onto each lane of a 4 – 12% Bis-Tris Nupage gel with MES SDS running buffer, according to the manufacturer's instructions. The gel was transferred to a PVDF membrane by electrophoresis at 100 V for 1 h. The membrane was blocked using PBS, 0.2% Tween 20 (PBS-T) and 5% nonfat milk at room temperature for 1 h. All antibodies were diluted in the same blocking buffer. The membrane was then probed with rabbit anti-p21 polyclonal antibody (1:1000 dilution), rabbit anti-p27 polyclonal antibody (1:1000 dilution), mouse anti-p53 monoclonal antibody (1:2000 dilution), and mouse anti-β-actin monoclonal antibody (1:5000 dilution), and then incubated for 1 h at room temperature. After incubation, the membrane was washed with PBS-T and incubated with horseradish peroxidase-conjugated goat anti-rabbit or mouse IgG (1:2000 dilution) for 2 h at room temperature. After washing with PBS-T, an ECL system was used to detect the proteins. Each assay was replicated in four independent experiments.

### Transfection of small interfering RNA (siRNA) into NHLF

p21, p27, p53 and control siRNAs were designed and synthesized by B-Bridge International Inc. (Sunnyvale, CA, USA). The p21, p27, p53 and negative control siRNA target sequences were 5'-CGUCAGAACCCAUGCGGCA-3', 5'-GGAGCAAUGCGCAGGAAUA-3', 5'-CUGGAAGACUCCAGUGGUA-3', and 5'-AUCCGCGCGAUAGUACGUA-3', respectively. NHLF were seeded into 6-cm dishes and incubated in DMEM with 10% FBS for 24 h, after which time they had reached about 60% confluence. After rinsing, the cells were incubated with liposome solution comprised of Opti-MEM medium, Lipofectamine 2000 (10 μl/ml), and siRNA (0 – 100 or 50 nM) with 10% FBS. After 8 h of incubation, the same amount of Opti-MEM medium containing 10% FBS was added to the dishes or plates and the incubation was continued for 16 h. After 24 h of transfection, the liposome solutions were replaced with DMEM containing 10% FBS. Next, the cells were harvested and seeded into 6-cm dishes or 96-well plates at a density of 6,000 cells/cm^2^, then cultured for 24 h in various concentrations of oxygen or with 100 μM DFX. After incubation, BrdU incorporation by the transfected cells was measured, and Western blot analysis was performed to confirm target gene silencing by each siRNA.

### Transient transfection of the p53 gene into NHLF

pCMV p53 (wild-type p53 genes), pCMV p53mt135 (dominant-negative mutants of p53) and pCMV β-galactosidase were purchased from Clontech (San Jose, CA, USA). All plasmids were purified using Qiagen plasmid midi and maxi kits. NHLF were seeded into 6-cm dishes and incubated in DMEM containing 10% FBS for 24 h, after which time they had reached about 70% confluence. After rinsing, the cells were incubated with 2.5 ml of liposome solution comprised of serum-free Opti-MEM medium, Lipofectamine 2000 (10 μl/plate), Lipofectamine-Plus reagent (40 μl/plate), and plasmid DNA (3 μg/plate). After 5 h of incubation, the same amount of Opti-MEM medium containing 20% FBS was added to the dishes and the incubation was continued for another 19 h. After 24 h of transfection, the cells were washed twice with PBS, and the liposome solution was replaced with DMEM containing 10% FBS. After 24 h incubation, protein expression of p21, p27 and p53 was examined by Western blot analysis. We assessed transfection efficacy by co-transfecting cells with pCMV β-galactosidase and determining the number of cells that stained blue with X-gal. Briefly, cells on collagen-coated membranes were fixed with 0.25% glutaraldehyde for 10 min, washed twice with PBS containing 1 mM MgCl_2_, and stained with 1 mg/ml X-gal in a solution of 1 mM MgCl_2_, 5 mM K_3_Fe(CN)_6_, and 5 mM K_4_Fe(CN)_6 _for 4 h. The transfection efficacy was routinely found to be more than 40%.

### Statistical analysis

Results are expressed as mean ± SE. Statistical analysis was performed using ANOVA with Bonferroni corrections for multiple comparisons. Comparisons were considered statistically significant at p < 0.05.

## Results

### Effect of hypoxia and DFX on NHLF proliferation

**Figure 1:**
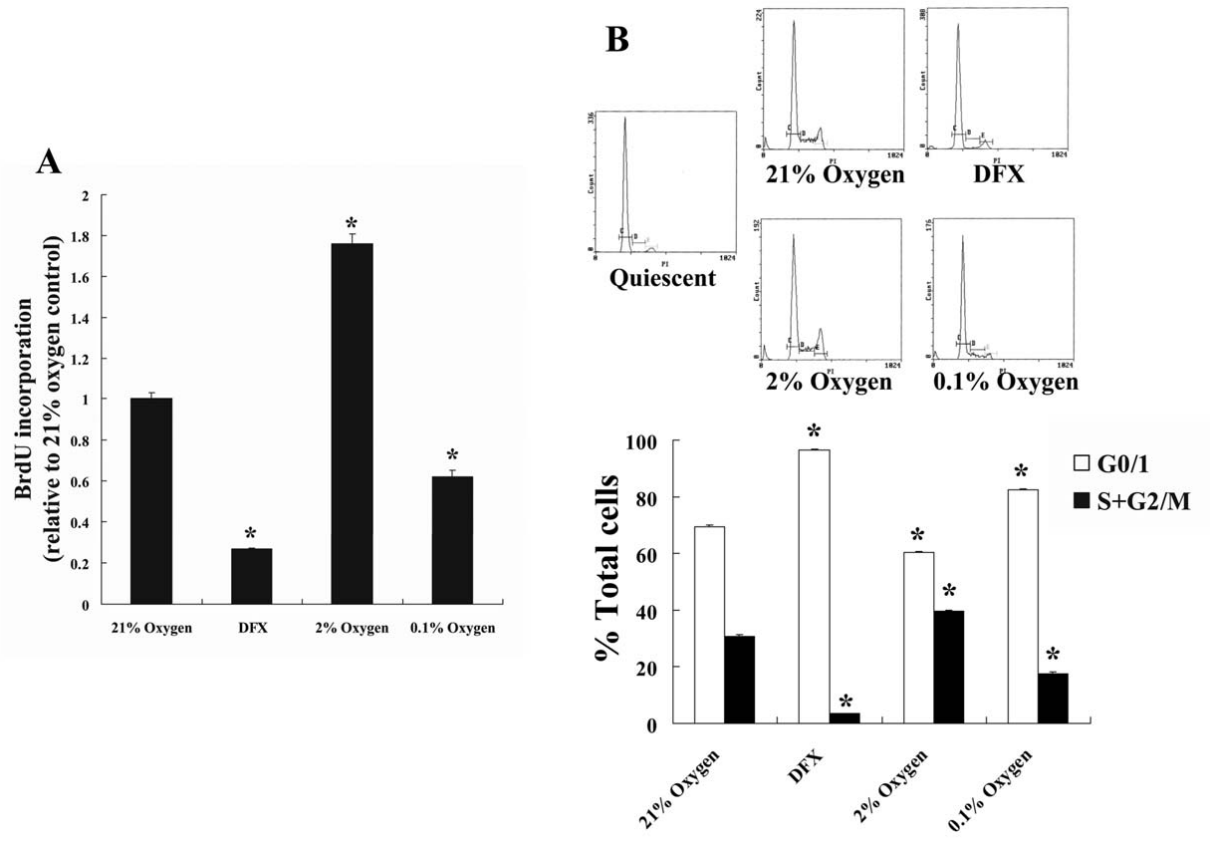
**Effect of hypoxia and DFX on NHLF proliferation**. A; Effects of hypoxia and DFX on BrdU incorporation in cultured NHLF: Cultured NHLF were exposed to 0.1% or 2% oxygen, or 100 μM DFX, in the presence of BrdU for 24 h. Severe hypoxia (0.1% oxygen) and DFX suppressed BrdU incorporation, but moderate hypoxia (2% oxygen) significantly enhanced incorporation. Data are expressed as mean ± SE (n = 6). *P < 0.05 versus the 21% oxygen control. B; Cell cycle analysis of NHLF exposed to hypoxia and DFX: Cultured NHLF (synchronized at G_0/1 _phases by serum depletion) were exposed to 0.1% or 2% oxygen, or 100 μM DFX, in the presence of 10% FBS. After 24 h, the cells were harvested and cell cycle analyses were performed using a flow cytometer with propidium iodide staining. Severe hypoxia and DFX significantly decreased the percentage of cells in the S plus G_2_/M phases, whereas moderate hypoxia had the opposite effect. Representative histograms are shown and the bar graph shows data expressed as mean ± SE (n = 4). Open bars, percentages of cells in the G_0/1 _phases; solid bars, percentages of cells in the S + G_2_/M phases. *P < 0.05 versus the 21% oxygen control.

Moderate hypoxia (2% oxygen) promoted the incorporation of BrdU in serum-stimulated NHLF, whereas severe hypoxia (0.1% oxygen) and 100 μM DFX suppressed BrdU incorporation (Fig. 1A). Cell cycle analysis using propidium iodide staining revealed that severe hypoxia and 100 μM DFX significantly decreased the percentage of cells in S plus G2/M phases, whereas moderate hypoxia had the opposite effect (Fig. 1B).

### Effect of hypoxia and DFX on expression of p21 and p53

**Figure 2:**
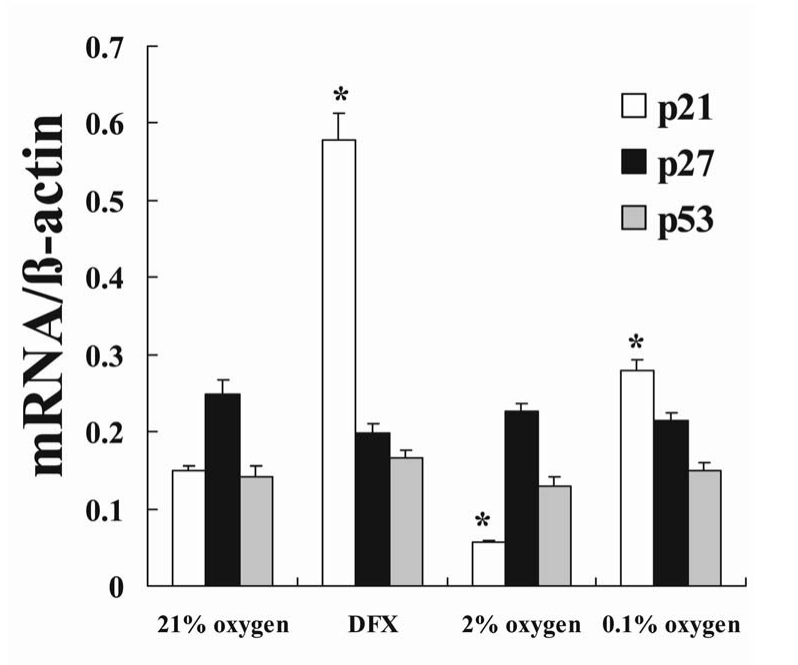
**Expression of p21 and p53 mRNA in NHLF exposed tohypoxia or DFX**. Cultured NHLF were exposed to 0.1% or 2% oxygen, or 100 μM DFX, for 24 h, then real-time RT-PCR analyses were performed. The amount of p21 mRNA was increased by severe hypoxia but decreased by moderate hypoxia. No significant changes were detected in the expression of p27 and p53 mRNA. The bar graph shows the ratios of p21 (open bars), p27 (solid bars), and p53 (shaded bars) mRNA relative to the amount of β-actin mRNA. Data are expressed as mean ± SE (n = 6). *P < 0.05 versus the 21% oxygen control.

Real-time RT-PCR showed that expression of p21 mRNA in NHLF decreased when cells were exposed to moderate hypoxia, whereas severe hypoxia and DFX both significantly increased the expression of p21 mRNA. In contrast, no significant changes in the expression of p27 and p53 mRNA were detected (Fig. 2).

**Figure 3:**
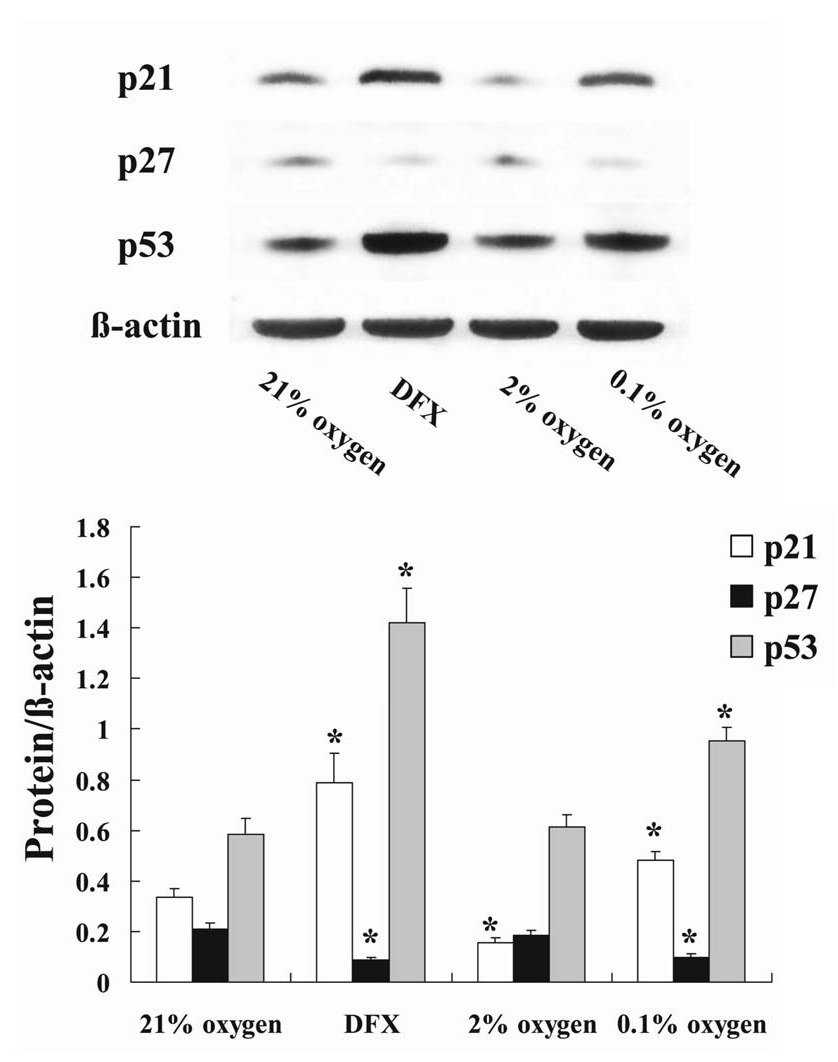
**Expression of p21, p27 and p53 protein in NHLF exposed to hypoxia or DFX**. Cultured NHLF were exposed to 0.1% or 2% oxygen, or 100 μM DFX, for 24 h, then Western blot analyses were performed. Expression of p21 was decreased by moderate hypoxia and increased by severe hypoxia and DFX. Expression of p53 was increased by severe hypoxia and DFX. Expression of p27 was decreased by severe hypoxia and DFX. The photomicrograph shown is a representative image from four similar experiments, and the bar graph shows the density ratios of p21 (open bars), p27 (solid bars), and p53 (shaded bars) protein bands relative to β-actin bands. Data are expressed as mean ± SE (n = 4). *P < 0.05 versus the 21% oxygen control.

Western blot analysis showed that p21 protein expression was decreased by moderate hypoxia, compatible with the results of mRNA expression analysis. Severe hypoxia and DFX both increased expression of p21 and p53 protein, and decreased expression of p27 protein (Fig. 3).

**Figure 4:**
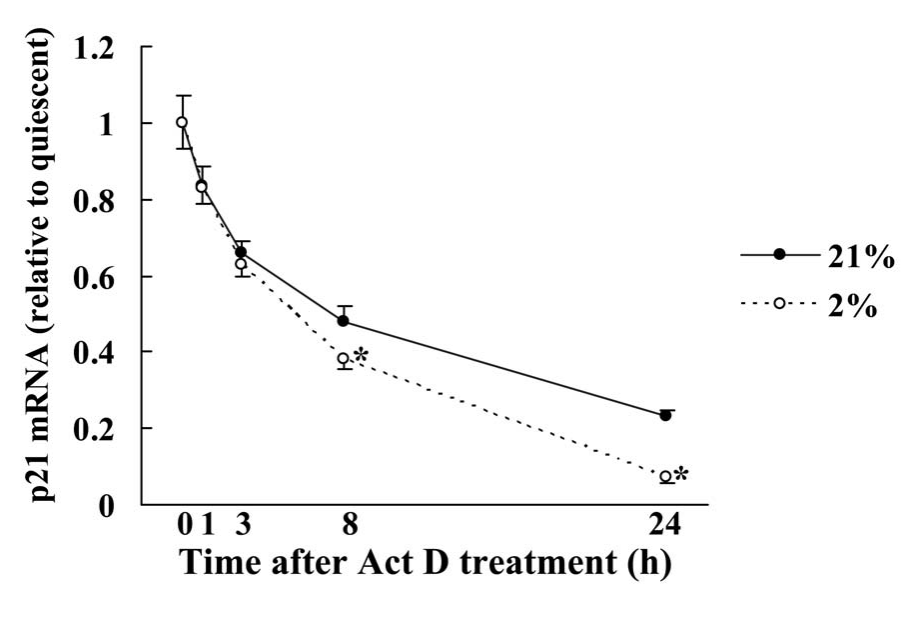
**Stability of p21 mRNA exposed to hypoxia in NHLF**. Cultured NHLF were exposed to 0.1% or 2% oxygen, or 100 μM DFX, and the stability of p21 mRNA was measured using RT-PCR after addition of 400 nM Act D. The stability of p21 mRNA significantly decreased after 8 and 24 h of moderate hypoxia relative to the 21% oxygen control. Data are percentages of each maximal mRNA expression. 21% oxygen control (open bars), 2% oxygen (solid bars), 0.1% oxygen (shaded bars), and DFX (diagonal bars). Data are expressed as mean ± SE (n = 6). *P < 0.05 versus the 21% oxygen control.

To confirm the effect of hypoxia on p21 mRNA expression, we assessed p21 mRNA stability during hypoxia using Act D. p21 mRNA stability was significantly decreased under moderately hypoxic conditions relative to the 21% oxygen control (Fig. 4).

### Effect of p53 gene induction and silencing in NHLF

**Figure 5:**
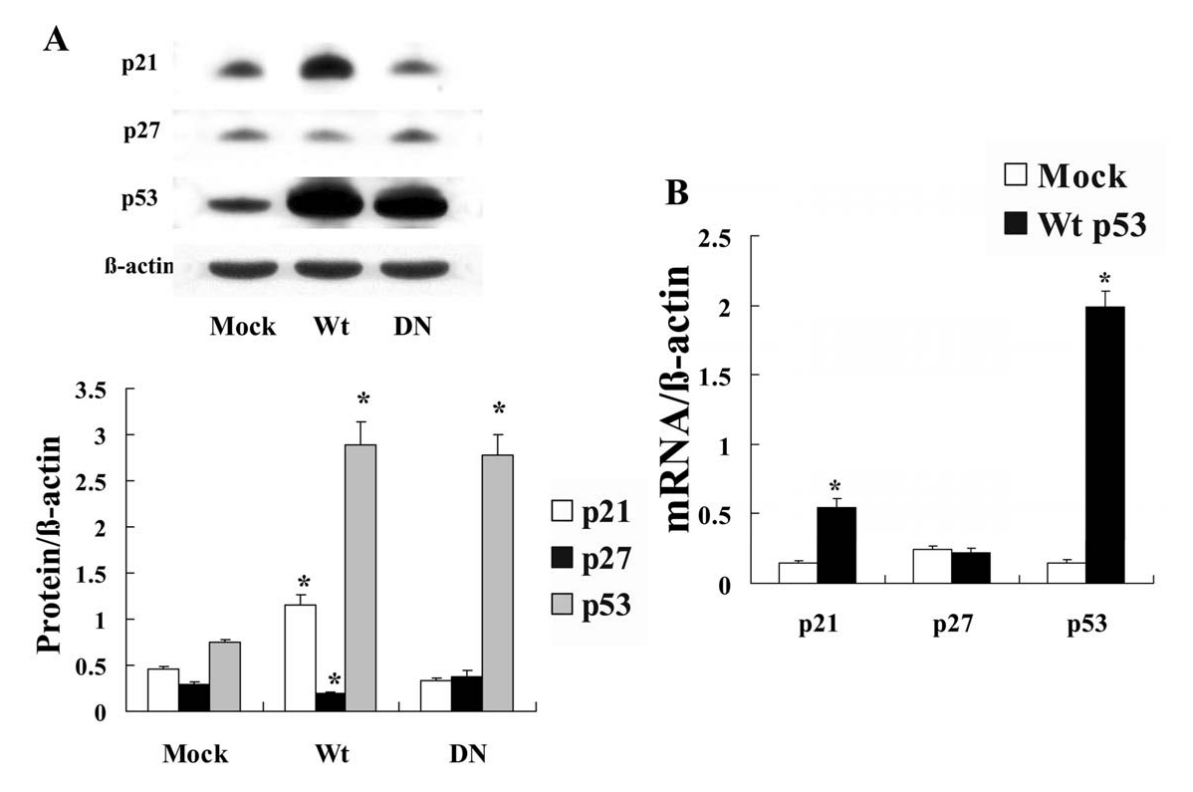
**Effect of p53 gene induction on p21, p27 and p53 expression in NHLF**. Expression of p21, p27 and p53 was examined in NHLF transfected with plasmids encoding wild-type (Wt) and dominant-negative mutation (DN) forms of the p53 gene, or an empty vector (Mock). A: Western blot analysis showed that Wt p53 transfection induced p21 protein expression and decreased p27 protein expression, whereas no significant changes in p21 and p27 protein expression were observed for the Mock- and DN-transfected cells. The photomicrograph shown is a representative image from four similar experiments, and the bar graphs show the density ratios of p21 (open bars), p27 (solid bars), and p53 (shaded bars) protein bands relative to β-actin bands. Data are expressed as mean ± SE (n = 4). *P < 0.05 versus Mock control. B: RT-PCR analysis showed that Wt p53 transfection induced p21 and p53 mRNA expression, whereas p27 mRNA was not affected. The bar graph shows the ratios of p21, p27 and p53 mRNA relative to the amount of β-actin mRNA. Open bars, Mock-transfected cells; solid bars, Wt p53-transfected cells. Data are expressed as mean ± SE (n = 6). *P < 0.05 versus Mock transfection.

**Figure 6:**
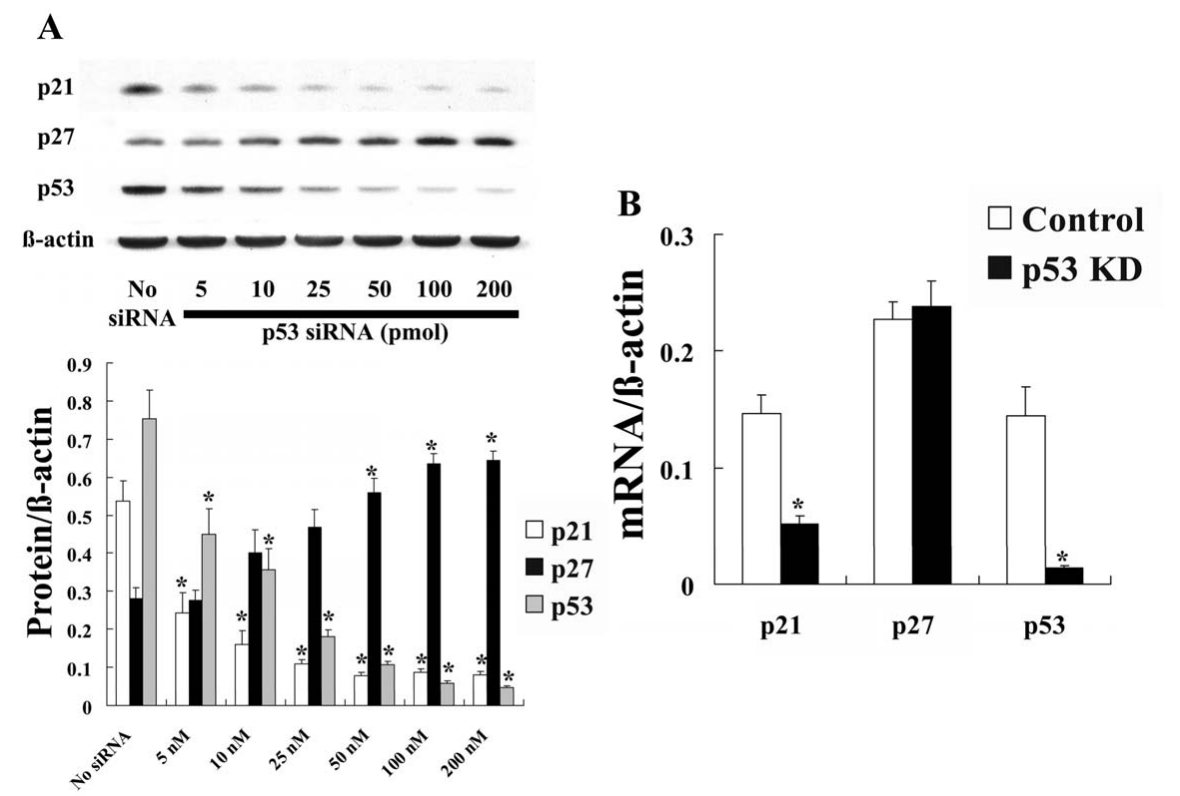
**Effect of p53 gene silencing on expression of p21, p27 and p53 in NHLF**. Expression of p21, p27 and p53 was examined in NHLF transfected with p53 siRNA (p53 KD) or control siRNA (Control). A: Western blot analysis showed that p53 KD dose-dependently decreased the amount of p53 and p21 protein and increased the amount of p27 protein. The photomicrograph shown is a representative image from four similar experiments, and the bar graphs show the density ratios of p21 (open bars), p27 (solid bars), and p53 (shaded bars) protein bands relative to β-actin bands. Data are expressed as mean ± SE (n = 4). *P < 0.05 versus control (no siRNA). B: RT-PCR analysis showed that p53 KD decreased the expression of p21 and p53 mRNA, whereas p27 mRNA was not affected relative to the control. Open bars, control siRNA-transfected cells; solid bars, p53 siRNA-transfected cells. Data are expressed as mean ± SE (n = 6). *P < 0.05 versus control siRNA transfection.

Transfection of cells with wild-type (Wt) and dominant-negative mutation (DN) forms of p53 induced expression of a massive amount of p53 protein. Only transfection with Wt p53 induced expression of p21 protein and decreased expression of p27 protein (Fig. 5A). RT-PCR analysis showed that transfection with Wt p53 induced p53 and p21 mRNA expression without affecting p27 mRNA expression (Fig. 5B). In contrast, transfection with p53 siRNA inhibited expression of p53 and p21 protein, and dose-dependently induced expression of p27 protein (Fig. 6A). p53 gene silencing significantly suppressed p53 and p21 mRNA expression without affecting p27 mRNA expression (Fig. 6B).

### Effect of hypoxia and DFX on proliferation in siRNA-transfected NHLF

**Figure 7:**
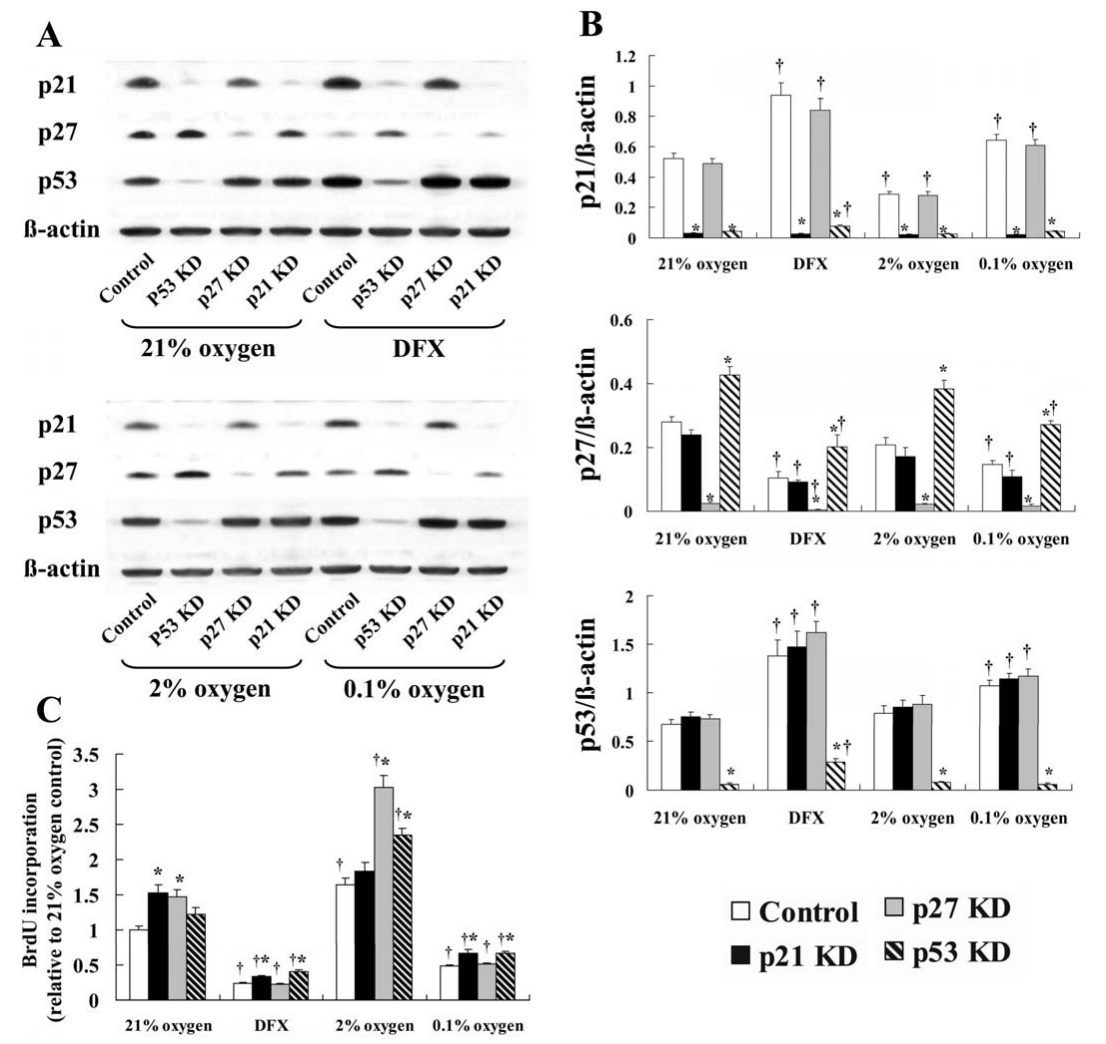
**Effect of hypoxia and DFX on proliferation in p21, p27 and p53 gene silenced NHLF**. NHLF transfected with control (Control), p21 (p21 KD), p27 (p27 KD), or p53 (p53 KD) siRNA were exposed to 0.1% or 2% oxygen, or 100 μM DFX. Western blotting was performed, and BrdU incorporation was measured. Transfection with p21, p27, and p53 siRNA significantly inhibited the expression of each target protein. p27 protein expression was increased by p53 KD transfection, and decreased by DFX and severe hypoxia. p21 protein expression was decreased by p53 KD transfection, decreased by moderate hypoxia, and increased by DFX and severe hypoxia. A: The photomicrographs shown are representative images from four similar experiments. B: The bar graphs show the density ratios of Control (open bars), p21 KD (solid bars), p27 KD (shaded bars), and p53 KD (diagonal bars) protein bands relative to β-actin bands. Data are expressed as mean ± SE (n = 4). *P < 0.05 versus control siRNA (Control). †P < 0.05 versus each siRNA in 21% oxygen. C: BrdU incorporation by p21 KD- and p27 KD-transfected cells was significantly increased compared with control cells in 21% oxygen. For moderate hypoxia, BrdU incorporation by p27 KD- and p53 KD-transfected cells was significantly increased compared with control cells, but p21 KD-transfected cells did not undergo any significant increase in proliferation. In severely hypoxic cells and cells exposed to DFX, BrdU incorporation was significantly increased in p21 KD- and p53 KD-transfected cells compared with control cells. The bar graph shows data expressed as mean ± SE (n = 6). Control siRNA (open bars), p21 KD (solid bars), p27 KD (shaded bars), and p53 KD (diagonal bars). *P < 0.05 versus control siRNA (Control). †P < 0.05 versus each siRNA in 21% oxygen.

Transfection of NHLF with p21, p27, and p53 siRNA significantly inhibited the expression of each target protein. p53 protein expression was significantly induced by DFX and severe hypoxia. p27 protein expression was significantly increased by p53 gene silencing, and decreased by DFX and severe hypoxia. p21 protein expression was significantly inhibited by p53 gene silencing, inhibited by moderate hypoxia, and induced by DFX and severe hypoxia (Fig. 7A, B). BrdU incorporation by p21 and p27 siRNA-transfected cells was significantly increased compared with the control siRNA-transfected cells in 21% oxygen. In moderate hypoxia, BrdU incorporation by p27 and p53 siRNA-transfected cells was significantly increased compared with control siRNA-transfected cells, whereas p21 siRNA-transfected cells did not undergo significantly more proliferation than control siRNA-transfected cells. In contrast, in severely hypoxic cells and cells exposed to DFX, BrdU incorporation was significantly increased by p21 and p53 siRNA transfection relative to control siRNA-transfected cells (Fig. 7C).

## Discussion

In the present study, we observed that moderate hypoxia (2% oxygen) enhanced the proliferation of NHLF, whereas severe hypoxia and DFX induced cell cycle arrest. We also demonstrated that both severe hypoxia and DFX cause cell cycle arrest via the p53-p21 pathway, but that moderate hypoxia enhances cell proliferation via a p21 pathway that is independent of p53 and p27. Further, our results suggest that p27 may be involved in compensating for p53 in serum-stimulated NHLF proliferation.

Increased levels of growth factors derived from the accumulation of HIF-1α are thought to regulate pulmonary arterial cell proliferation under hypoxic conditions, given that partial HIF-1α deficiency decreases the remodeling of pulmonary arterioles in animals exposed to chronic hypoxia [37]. Although HIF-1α regulates various transcriptional genes encoding angiogenic factors, severe hypoxia and iron depletion induce cell growth arrest. Our finding that severe hypoxia (0.1% oxygen) suppresses nucleotide synthesis is in line with the findings of previous studies in which several tumor cell lines were cultured under hypoxic conditions or with iron chelators [16,17]. In other studies, it has been found that moderate hypoxia (1 – 5% oxygen) enhances the proliferation of rat and bovine PASMC, airway-smooth muscle cells, lung fibroblasts and mesangial cells [3,18,19,38]. Our findings that DNA synthesis is increased during moderate hypoxia, and that the NHLF cell cycle progresses more quickly under hypoxic than normoxic conditions, are also compatible with previous findings.

There are some discrepancies regarding serum stimulation when we compare our results using NHLF and previous data obtained with human pulmonary artery fibroblasts which proliferate in the face of hypoxia and low levels of serum stimulation [39-41]. We do not have a complete answer for this discrepancy at this time. However, several papers have shown that normal fibroblasts (non-pulmonary artery fibroblasts) exposed to hypoxia need relatively high serum concentrations for posttranscriptional processing or cell proliferation [7,42], suggesting that pulmonary artery fibroblasts have a greater proliferative potential than normal fibroblasts. In addition, we assume that several CDK inhibitors associated with replicative senescence, which has been known to be associated with an increase in expression of p16, p21, and p19 [43,44], may play a role in this discrepancy. Further, serum depletion causes cell cycle arrest and an accumulation of p27 protein, and this degradation is enhanced by serum stimulation and hypoxia [45]. Taken together, we speculate that this increase of CDK inhibitors likely influences cell growth and reactions to serum stimulation during hypoxia, and our cells require relatively high levels of serum to grow compared to the pulmonary artery fibroblasts. However, further studies will be necessary to confirm this hypothesis.

p21 has been shown to regulate cell cycle progression through either a p53-dependent or -independent pathway [26,27,46,47]. Under conditions of moderate hypoxia, we observed decreased p21 expression and increased DNA synthesis with progress of the cell cycle in NHLF. In these cells, however, p53 protein was not affected, and decreased p21 stability was observed. These results indicate that the decrease in p21 expression in moderate hypoxia is independent of the p53 pathway, and is probably induced by a decrease in the stability of p21 mRNA. The precise molecular mechanism responsible for p53-independent p21 mRNA instability after exposure to moderate hypoxia remains unclear. However, Esposio et al. found that p21 protects cells from oxidative stress by inhibiting DNA replication, and that oxidative stress stabilizes p21 mRNA through mitogen-activated protein kinase (MAPK) [48,49]. In addition, hyperoxic conditions and application of hyperbaric oxygen induce p21 protein independently of p53 protein and lung injury caused by oxidative stress [27]. These observations may explain why a p53-independent decrease in p21 was observed in moderately hypoxic conditions. The decrease in p21 and cell proliferation during moderate hypoxia could be due to the secondary effects of decreased oxidative stress and release of free oxygen radicals. However, further studies are essential in order to determine the nature of the hypoxic regulation of p21.

p53 protein expression must ordinarily rely on post-transcriptional events, given that p53 protein is a very short-lived protein owing to fast proteasomal degradation, and that stabilization of the protein in response to a variety of stresses, including hypoxic stress, has been found to evoke a rapid increase in p53 levels [50]. In the present study we found that p53 protein expression was increased after severe hypoxic exposure and incubation with DFX, and that p21 mRNA and protein expression also occurred. However, in several previous studies using tumor cell lines with the wild-type p53 gene, p53 accumulation caused by iron chelators was found to induce p21 mRNA, but p21 protein was not expressed in response to inhibition of p21 translation [16,17,32]. Fukuchi et al. reported that p21 protein accumulation caused by iron chelators was dependent on inhibition of the proteasome [16], and Le et al. reported that p21 translation in the presence of DFX required iron, since this translational disorder could be reversed by the iron donor ferric ammonium citrate [34]. This discrepancy between our results and those of previous studies could be explained by differences in the cell lines used, because different cell lines may have different hypoxic tolerances relative to each proteasomal activity or capacity for iron content. However, the precise mechanism underlying this discrepancy is unknown and the matter requires further investigation.

Mutations in p53 and reduced expression of p27 are frequently observed in many human cancer cells, and reduced expression of p27 is a common feature in cancers with mutations in p53 [51-53]. Tumor development in p53 mutant mice was also found to be accelerated by loss of only a single allele of p27 [52]. These findings indicate that p27 is an important cell cycle regulator in cells that have a mutated p53 gene. In the present study, gene silencing of p53 increased p27 protein expression, and gene silencing of p27 could not suppress the increase in moderate hypoxia-induced DNA synthesis. p27 protein expression was not affected by mild hypoxia in our experiments. On the contrary, p21 protein expression in p27 gene silenced NHLF was significantly decreased in mild hypoxia. These results indicate that p27 gene silencing could not suppress the growth of hypoxic NHLF because the regulation of the p21 protein, which may have a critical role in mild hypoxic proliferation, was still active. Surprisingly, silencing of the p53 gene did not induce a significant increase in DNA synthesis at ambient oxygen concentrations, whereas p21 expression was significantly suppressed in these cells and silencing of the p21 gene had a marked effect on proliferation. Given these results, we conclude that p27 does not play a central role in oxygen-dependent NHLF proliferation, but does play a role in compensating for p53-suppressed proliferation in NHLF. Wu et al. reported that in serum-stimulated fibroblasts from p53 knock-out mice, p21 could not be activated, but p27 protein could be induced without a decrease in cyclin A expression [54]. This compensative role of p27 in the loss of the p53 protein is in line with the results of our study. In addition, our results regarding p21 and p53 gene silencing also suggest that accumulation of p53 during hypoxia or DFX treatment plays a critical role in cell cycle regulation via the p53-p21 pathway.

## Conclusion

We showed that decreased oxygen levels increased NHLF proliferation but very low oxygen levels caused cell cycle arrest. Decreased expression of p21 protein may play a p53-independent role during hypoxia-induced NHLF proliferation, but hypoxic cell cycle arrest is regulated by p21 induction by hypoxic accumulation of p53. p27 may have a compensatory role for the p53 pathway. Hypoxic pulmonary fibroblast proliferation may contribute to the progression of hypoxia-associated fibroblastic lung diseases and hypoxic pulmonary remodeling. We believe that a better understanding of the cellular mechanisms in hypoxic NHLF will lead to improved modes of therapy for hypoxia-associated changes in lung tissue structure and aid in the remodeling of the lung.

## Competing interests

The authors declare that they have no competing interests.

## Authors' contributions

SM conceived the ideas investigated in this study, carried out the laboratory measurements and drafted the manuscript. HB and NV participated in the revision of the manuscript. YU and MK participated in the laboratory measurements and data analyses. SA and IM participated in the design of the study. TI supervised the study and was involved in writing the manuscript. All authors read and approved the final manuscript.
